# Commentary about the paper: “Overview of clinical forensic services in various countries of European Union”

**DOI:** 10.1080/20961790.2019.1659475

**Published:** 2019-10-30

**Authors:** Bertrand Ludes

**Affiliations:** University of Paris, Paris, France

The article about the “Overview of clinical forensic services in various countries of European Union” [1] is an important milestone in the field of clinical forensic medicine in so far that one of the main proposals would be to set up a Clinical Forensic Network for Europe. This network could be held under the umbrella of the European Council of Legal Medicine.

The project described in this paper was submitted under the “Joint Justice & Daphne call–Actions grants to support national or transnational projects to enhance the rights of victims of crime/victims of violence-JUST/2015/SPOB/AG/VIC” and is co-funded by the Justice Programme of the European Union (EU) [2]. The JUST_e_U! project started in February 2017 for a period of 2 years and addresses the access to specialist support services and specifically to clinical forensic examinations [3]. The medical care of victims of sexual and/or physical violence consists in a clinical forensic examination during which injuries are documented in detail on a documentation form as well as pictures taken of the lesions and collection of trace evidences which are stored in adequate conditions. These evidential findings can then be used in upcoming legal proceedings [4].

The project consortium involves the Institute of Forensic and Traffic Medicine at the University Hospital Heidelberg and the Institute for Forensic Medicine at the Hannover Medical School in Germany, the Department of Medical and Surgical Specialties, Radiological Sciences, and Public Health at the Università degli Studi di Brescia in Italy, the Department of Forensic Medicine at the Faculty of Medicine in Hradec Králové and the Faculty of Law at Palacký University Olomouc in the Czech Republic. The Ludwig Boltzmann Institute for Clinical Forensic Imaging was the project leader. Finally, it was possible for this consortium to gather forensic expertise from 11 European countries: Austria, Croatia, the Czech Republic, Germany, Ireland, Italy, Luxembourg, Portugal, Romania, Slovakia and Slovenia.

One main part of the project focuses on dissemination and awareness-raising activities to expand the knowledge on the relevance of an access to clinical forensic examinations for victim support in the public as well as amongst experts.

The goals of the JUST_e_U! were to discuss a future Clinical Forensic Network for Europe (CFN Europe) and a European-wide minimum standard for clinical forensic examinations.

The starting point for the discussions was to create two questionnaires, one was dedicated to analyse the legal framework concerning clinical forensic examinations, the second was concerning the availability of clinical forensic service offers in the different countries.

The results showed that a European-wide network could offer many advantages for victims and for medical staff and this paper, as the different guidelines published by the European Council of Legal Medicine [5,6], represent a strong argument on the European level to achieve the implementation of guidelines and standards as well as the funding for the examination services. Furthermore, victims should have equal rights and receive equal support and protection in all the EU countries. It must be stressed out that the evidential findings within a clinical forensic examination have a higher value of evidence in court. The authors stated that the setting up of a CFN Europe could allow victims to have an access up to date and easily gain information on how to contact respective where to find a specialist for a clinical forensic examination. Another advantage of a CFN Europe would be that the public would have more awareness for the issue of domestic and sexual violence in the public, which could encourage victims to come forward and report their cases. The CFN Europe could also be the body where experts and medical staffs and paramedics could exchange on their experience, on mutual learning and research opportunities with international experts. Furthermore, the CFN Europe could be a network proposing training for medical staff and other occupational groups who are in close contact with victims of physical and/or sexual violence in order to secure and adequately store forensic findings.

The highlights of this paper are that clinical forensic services should be built on “three pillars: ‘Awareness raising in public’, ‘Support by state/politics’ and ‘Training’”. The clinical forensic units must have, to guarantee adequate support for victims of all forms of violence, an availability of an on-call service, provided 24/7.

The JUST_e_U! project could be seen as the starting point to push forward clinical forensic medicine on the European level, but this work should be extended to other countries of the EU that did not participate in the study with the scientific support of the European Council of Legal Medicine.

Bertrand Ludes *University of Paris, Paris,
France* bertrand.ludes@parisdescartes.fr
